# Association between Frequency of Toothbrushing and Metabolic Syndrome among Adolescents: A 5-Year Follow-Up Study

**DOI:** 10.3390/ijerph19010508

**Published:** 2022-01-03

**Authors:** Jagan Kumar Baskaradoss, Mary Tavares, Fahd Al-Mulla, Ebaa Al-Ozairi, Mohamed Abu-Farha, Saadoun Bin-Hasan, Aishah Alsumait, Sriraman Devarajan, Hend Alqaderi

**Affiliations:** 1 Department of Developmental and Preventive Sciences, Faculty of Dentistry, Kuwait University, Kuwait City 13110, Kuwait; 2 The Forsyth Institute, 245 First Street, Cambridge, MA 02142, USA; mtavares@forsyth.org; 3 Department of Genetics and Bioinformatics, Dasman Diabetes Institute, Dasman 15462, Kuwait; fahd.almulla@dasmaninstitute.org (F.A.-M.); ebaa.alozairi@dasmaninstitute.org (E.A.-O.); mohamed.abufarha@dasmaninstitute.org (M.A.-F.); Saadoun.binhasan@icloud.com (S.B.-H.); sriraman.devarajan@dasmaninstitute.org (S.D.); hend_alqaderi@hsdm.harvard.edu (H.A.); 4 Department of Medicine, Faculty of Medicine, Kuwait University, Kuwait City 13110, Kuwait; 5 Farwaniya Hospital, Ministry of Health, Farwaniya 92400, Kuwait; 6 Kuwait School Oral Health Program, Ministry of Health, P.O. Box No. 5338, Salmiya 22064, Kuwait; aalsumait@hotmail.com; 7 Department of Oral Health Policy & Epidemiology, Harvard School of Dental Medicine, Boston, MA 02115, USA

**Keywords:** biomarkers, children, C-reactive protein, diet, inflammatory, oral hygiene

## Abstract

This study longitudinally examines the relationship between the frequency of toothbrushing and the development of selected components of metabolic syndrome (MetS), along with the potential role of salivary biomarkers in this relationship. In 2014, 6317 12-year-old children underwent health examinations (T1), of which, 348 children participated in the second stage of data collection in 2019 (T2). The association between the change in the metabolic status during the 5-year follow-up examination (between T1 and T2) and frequency of toothbrushing was assessed using multinomial logistic regression analyses. At T2, healthy adolescents had significantly higher odds of toothbrushing twice or more daily compared with adolescents with components of MetS (OR = 1.99, 95% CI 1.15–3.45). Adolescents who were healthy at T1 but developed components of MetS at T2, had significantly higher frequencies of dining-out compared with adolescents with components of MetS at both T1 and T2 (OR = 0.09, 95% CI 0.02 to 0.49). Adolescents who were ‘healthy’ at both T1 and T2 had significantly (*p* < 0.05) lower levels of C-reactive protein (T2), insulin (T1 and T2), interleukin-6 (T1) and adiponectin (T1) compared with adolescents who had components of MetS. Toothbrushing and frequency of dining-out were associated with the presence of MetS components.

## 1. Introduction

Metabolic syndrome (MetS) is a cluster of interrelated patient-level factors that increases the risk for development of cardiovascular disease and type-2 diabetes. Many international health groups, such as the World Health Organization (WHO), the American Heart Association (AHA) and the International Diabetes Federation (IDF), have developed criteria for defining MetS, which is characterized by obesity, abnormal glucose tolerance, hypertension, high triglycerides and reduced high-density lipoprotein cholesterol (HDLC) [1]. In children and adolescents, however, there is no clear consensus on the definition of MetS [2].

The prevalence of obesity and hypertension in children and adolescents has increased significantly in the last two decades [3]. The most common component of MetS seen in children and adolescents, is obesity. In the United States, the percentage of children who are overweight has tripled in the last two decades to current estimates of 15% among those 6 to 19 years of age [4]. Increasing rates of obesity among the younger population is a public health concern due to the long-term negative effects of their overall health and well-being. Hypertension, on the other hand, is one of the most important predictors of cardiovascular disease mortality [5]. Although, when compared to adults, the prevalence of primary hypertension is much lower in the younger age group, childhood obesity has been shown to be strongly associated with both hypertension and pre-hypertension [6].

MetS is believed to develop from a pro-inflammatory state due to the effects of insulin resistance and periodontitis being associated with systemic inflammatory response and low-grade systemic inflammation [7]. Common risk factors link oral and systemic diseases, and their interconnection is well documented [8]. Existing evidence supports the plausible relationship between MetS and oral health [9,10]. A large study among middle-aged Japanese adults found that metabolic syndrome was associated with the incidence of tooth loss [11]. Several studies have explored the relationship between periodontitis and MetS and common inflammatory pathways may mediate this relationship [12,13]. Studies have reported several biomarkers that significantly correlate with metabolic syndrome [14] and periodontitis [15]. Interestingly, Tanaka et al. [16] reported that the risk of developing MetS decreased with higher frequency of daily toothbrushing, regardless of the periodontal status, indicating a direct relationship. Kobayashi et al. [17] also found that the frequency of toothbrushing was related to a lower prevalence and incidence of MetS. More recently, a longitudinal study among adults concluded that toothbrushing habits are associated with the development of obesity and hyperglycemia [18].

To date, the role of salivary biomarkers in the relationship between toothbrushing and MetS remains uninvestigated among adolescents. With this background, the aim of this study is to examine the association between frequency of toothbrushing and the development of selected components of MetS (obesity and hypertension), along with the potential role of salivary biomarkers in this relationship.

## 2. Materials and Methods

### 2.1. Ethical Considerations

Ethical approval for this study was obtained from the Ministry of Health in Kuwait, the Dasman Diabetes Institute Human Ethical Review Committee and the Forsyth Institutional Review Board. Informed consent was obtained from the study participants, and the study is reported in accordance with the guidelines of Strengthening the Reporting of Observational Studies in Epidemiology (STROBE) for observational clinical studies [19].

### 2.2. Study Population

This study was part of a larger project entitled, “Kuwait Healthy Lifestyle Study (KHLS)”, which is an ongoing collaborative research project, jointly conducted by the Dasman Diabetes Institute (DDI) in Kuwait and the Forsyth Institute in the United States. Sample size calculation was performed based on the protocol proposed by Schwab for multinomial logistic regression analysis [20], which allowed the inclusion of 10 cases for each combination of independent variables. In this study, the final model considered a maximum of 15 variables and a correction factor of 2.0 was applied to increase the accuracy. Therefore, the minimum required sample was 300 subjects.

A detailed description of the overall study design has been previously described [21,22,23,24]. In brief, 6317 12-year-old children from 138 public schools, representing all the six governorates in Kuwait were examined in 2014 (T1). The distribution of participants across the six governorates of Kuwait was approximately equal, representing all the social classes and ethnicities among the Kuwaiti population. In 2019 (T2), a two-stage cluster sampling technique was used for the selection of the sample. For the first stage, two governorates were randomly selected and a list of schools of children who participated in the T1 phase from these governorates was compiled. To match the a priori sample estimates, the decision was made to randomly select 19 schools, representing about 10% of the schools of children who participated in T1. Permission to conduct the study in each of the selected schools was obtained from the concerned school authorities. In the second stage, all the students who participated in T1 from the selected schools (N = 550) were invited to participate in the study. Informed consent was obtained from parents (or guardians) for T1 participants and from the students themselves at T2. Consenting students were provided with the necessary instructions, including the time and date of the proposed visit by the research team. Students were instructed to avoid taking any food 12 h prior to the scheduled appointment. Reminders were sent to the students 24 h prior to the appointment date to reinforce the instructions.

### 2.3. Data Collection

Children who consented to participate in the study were examined within the school premises by two calibrated and trained teams, as previously described [24,25]. The research team met the students as soon as they reached the school campus, prior to the start of their morning session. A structured interview schedule gathered information about the participants regarding their demographic variables, dental history, oral hygiene behavior, dietary characteristics and physical activity patterns. This was followed by anthropometric evaluation, blood pressure assessment, oral examination, saliva sample collection and nutrition analysis. The information was directly entered into a programmed iPad (Apple, Cupertino, CA, USA)

### 2.4. Saliva Collection

After the subjects rinsed their mouths with 15 mL of water, approximately 4 mL of unstimulated saliva was collected. The samples were centrifuged at 2800 rpm for 20 min at 4 °C, after which the supernatants were transferred to screw-cap, 2D-barcoded storage tubes (Matrix^®^ 2D Barcoded ScrewTop^®^ storage tubes, Thermo Fisher Scientific Inc., Hudson, NH, USA), which were read by a barcode reader (Thermo Scientific VisionMate^®^ ST Barcode Reader, Thermo Fisher Scientific Inc., Hudson, NH, USA) by which participant number was related to sample number. The saliva samples were stored at −80 °C until assayed. 

### 2.5. Multiplex Analysis of Salivary Biomarkers

All assays were performed as previously described [21]. In brief, the frozen samples were transported to the Forsyth Institute on temperature-monitored dry ice. Samples were thawed at 4 °C overnight and kept on ice throughout the assay procedure. Insulin, C-reactive protein (CRP), adiponectin, leptin, interleukin (IL) -6, IL-8, IL-10, monocyte chemotactic protein (MCP) and vascular endothelial growth factor (VEGF) were measured in 25 μL of saliva samples using multiplex magnetic bead panels on a Luminex 200™ system (Luminex, Austin, TX, USA) at the Forsyth Institute Multiplex Core Facility (Cambridge, MA, USA). Results were evaluated using Bio-Plex Manager™ (Version 5.0; Bio-Rad, Hercules, CA, USA).

### 2.6. Data Transformation

In this study, adolescents were classified as at risk for MetS if they had one or more of the following conditions: obesity, hypertension. Obesity was the dependent binary variable representing obese vs. non-obese adolescents (WHO criteria [26]) in our analyses. Height was measured with a stadiometer, weight was measured with a calibrated digital scale, and body mass index (BMI)-for-sex/age z score (BMIz) was calculated by computer using WHO guidelines [26]. Hypertension is defined as having either systolic or diastolic blood pressure in the 95th percentile with respect to height, age and sex, using the IDF guidelines [27].

The outcome variable was defined as the change in metabolic status at T1 and T2. Based on the change in metabolic status, the participants were grouped into four categories: Group 1 participants were healthy at T1 and remained healthy at T2; Group 2 participants were healthy at T1 and became obese/hypertensive at T2; Group 3 participants were obese/hypertensive at T1 and became healthy at T2; and Group 4 participants were obese/hypertensive at both T1 and T2.

Twenty-six dietary characteristics, collected by our questionnaire for each child, were first grouped into eight eating pattern analyses. To further reduce the number of dimensions in the eight eating categories, we conducted principal component analysis (PCA) to derive dietary patterns. After considering the scree plot derived from the PCA, we decided to retain the first principal component, which was named “dine-out pattern”. Considering the variability in the eating patterns, study participants were not rigidly classified as “following” or “not following” a given dietary pattern. Rather, they were ranked into percentiles by calculating the dietary pattern scores for each participant according to how closely their eating styles aligned with the dietary pattern. The dietary pattern score was constructed by summing the observed values of eight dietary categories, which were weighted by their factor loadings derived from our PCA. The “dine-out pattern” score was categorized into quartiles: 1st quartile—‘rarely’; 2nd quartile—‘sometimes’; 3rd quartile—‘often’; and 4th quartile—‘frequently’.

The physical activity pattern was assessed using a modified version of the previously validated questionnaire from the Arab Teens Lifestyle Survey (ATLS) [28]. The questionnaire was designed to collect information on frequency, duration and intensity of a variety of light-, moderate- and vigorous-intensity physical activities during a typical week. Physical activities were assigned metabolic equivalent of task (MET) values based on the compendium of physical activity for youth [29]. The median MET values were taken to dichotomize the physical activity patterns into “good” (≥median score) and “poor” (<median score). Toothbrushing frequency was categorized as brushing teeth once per day or less and twice per day or more. The percentage total of untreated decayed (D and d) and filled (F and f) teeth were used as a measure of dental caries experience and the percentage of untreated decay alone was used to measure the severity of the caries [23].

### 2.7. Statistical Analysis

Distribution of the outcome variable between the groups was explored for normality using the Kolmogorov–Smirnov and Shapiro–Wilk tests. The chi-square test was used for nominal qualitative variables (socio-demographic and dental characteristics) and the *t*-test was used for comparing continuous data between two groups for variables with normal distributions. To account for the skewed distribution of the salivary biomarkers, pairwise comparisons using the Wilcoxon rank sum test were used to assess the difference in the median scores between the T1 and T2 visits. The association between the change in metabolic status (dependent variable) during the 5-year follow-up examination (between T1 and T2) and frequency of toothbrushing (independent variable) was assessed using multinomial logistic regression analyses, expressed in odds ratio’s (OR’s) with 95% confidence intervals (CI’s). Multinomial logistic regression was conducted to determine the factors that contributed to the change in the metabolic status of the participants. Three different regression models were constructed. The first model was adjusted for gender, diet and physical activity. The second model was additionally adjusted for maternal/paternal education, maternal/paternal diabetic status and medical history. The third model was additionally adjusted for dental characteristics (the number of decayed teeth, last dental visit, dental pain in the last year and embarrassment due to teeth problems). A statistical significance level of 5% was adopted. All statistical analyses were carried out using SPSS 27.0 (IBM Corp., Armonk, NY, USA).

## 3. Results

A total of 348 children (males = 168 and females = 180) participated in this study. The mean age of respondents at T1 was 12.06 years (males = 12.13; females = 11.99) and at T2 it was 17.04 years (males = 17.11; females = 16.97). The mean BMI at T1 was 22.81 (SD = 5.81; range = 32.03) and at T2 it was 27.01 (SD = 7.61; range = 37.33). The mean BMI scores were not significantly different between males and females at both T1 (22.58 ± 6.01 and 23.03 ± 5.63, respectively) and T2 (26.86 ± 7.61 and 27.14 ± 7.63, respectively). A similar proportion of males and females were categorized as obese (60.7% and 59.4%, respectively).

Baseline characteristics, stratified by toothbrushing frequency, are summarized in Table 1. More than half of the participants (53.4%) reported that they brushed twice daily or more. A significantly higher percentage (60.6%) of female subjects reported to brush twice daily or more compared with males (45.8%) (*p* = 0.01). Parent educational level and existing medical conditions were not significantly different between the two toothbrushing groups (*p* > 0.05). Compared with participants with a toothbrushing frequency of ≤1 time/day, those who brushed ≥2 times/day had significantly higher physical activity patterns and there were also fewer obese individuals at T2 (*p* < 0.05). No significant difference in the ‘dining-out’ dietary pattern was observed between the toothbrushing groups. About one-fourth (23.9%) of the study participants belonged to Group 1 (were and remained healthy) and more than half (51.4%) of the participants belonged to Group 4 (were and remained obese/hypertensive), at T1 and T2 visits. About 7% of the participants were in Group 2 and about 18% belonged to Group 3. A significant difference was observed in the change in metabolic characteristics from T1 to T2 and the frequency of toothbrushing (*p* = 0.03). A higher percentage of individuals in Group 1 (62.7%) regularly brushed their teeth compared with those in Group 4 (45.8%). The incidence of hypertension in the 5-year follow-up period (between T1 and T2) for the sample was 11.7%, and for obesity it was 13.7%.

The percentage of caries experience (D/d + F/f) was 12.96 (SD = 0.66) and the severity of caries (D/d only) was 8.90 (SD = 0.55). Approximately half (51.7%) of the sample had a dental visit within six months of the study. No significant difference between the groups was observed for any of the other dental characteristics (Table 2).

Table 3 presents the distribution of the salivary biomarkers stratified by brushing frequency. There was no significant difference in the salivary biomarkers between the toothbrushing groups.

Table 4 presents the median values of the salivary biomarkers at T1 and T2 stratified by MetS status. A significant decrease (*p* < 0.05) was observed for the overall levels of IL-6, IL-8, adiponectin and VEGF at the T2 visit compared with that of T1. Group 1 subjects had significantly (*p* < 0.05) lower levels of CRP (T2), insulin (T1 and T2), IL-6 (T1) and adiponectin (T1) compared with Group 4 subjects.

Table 5 presents the results of the multinomial logistic regression analysis. Group 4 were assigned as the reference group. In Model 1, compared to Group 4 (members were and remained obese/hypertensive), Group 1 (members were and remained healthy) had significantly higher odds of toothbrushing twice or more daily (OR 1.99, 95% CI 1.15–3.45). The significant association was observed even after adjusting for the covariates in Model 2 (OR 2.05, 95% CI 1.15 to 3.65) and Model 3 (OR 1.99, 95% CI 1.06 to 3.72). Compared to Group 4, Group 3 had significantly higher odds of toothbrushing twice or more daily in Model 2 (OR 2.16, 95% CI 1.13–4.14) and Model 3 (OR 2.02, 95% CI 1.03–3.99). For the frequency of dining-out, Group 2 had significantly higher odds of frequently dining out in all the three models (Model 1 = OR 0.14, 95% CI 0.03 to 0.68; Model 2 = OR 0.11, 95% CI 0.02 to 0.56 and Model 3 = OR 0.09, 95% CI 0.02 to 0.49).

## 4. Discussion

This study demonstrated that subjects with MetS brushed their teeth less frequently than subjects without MetS. Although a few studies have observed the association between toothbrushing and MetS in adult populations, this relationship is not well characterized among children or adolescents.

Since there is still no universally accepted definition of MetS in children and adolescents, most studies on the pediatric population, therefore, have used definitions that are adapted from adult standards with the use of gender- and age-dependent normal values [30]. An earlier study [31] in the same cohort, reported good predictive capacity by assessing only two risk factors (obesity and high blood pressure). This study utilized the same criterion for estimating the emerging state of MetS in children. Using the combination of obesity and high blood pressure to determine the clinical phenotype for MetS has been employed in the past as well. Campbell et al. [32] reported that both obesity and high BP showed greater expression of lipid and glucose abnormalities, higher urinary albumin excretion and a greater prevalence of prediabetes, undetected type-2 diabetes mellitus and insulin resistance syndrome.

There is a strong body of evidence showing that oral health is associated with systemic diseases such as type-2 diabetes mellitus, chronic kidney disease, rheumatoid arthritis, cognitive impairment and MetS [33]. To maintain good oral health, the American Dental Association (ADA) and other public health agencies recommend brushing twice a day with fluoridated toothpaste and interdental cleaning once a day [34]. Good oral hygiene reduces the risk of caries, gingival inflammation and periodontitis [35]. A Japanese study found that the frequency of toothbrushing was the most predictable indicator of general health behaviors [36]. A low frequency of toothbrushing was associated with a higher prevalence of diabetes mellitus [37], dyslipidemia [37], cardiovascular events [38], hypertriglyceridemia [17] and MetS [16].

The biological plausibility of the association between toothbrushing and MetS is not yet clearly established. One possible explanation for this association is that regular toothbrushing helps to reduce gingival inflammation, which decreases chronic low-grade systemic inflammation that can contribute to the development of MetS [39]. However, in this study, salivary-biomarker analyses revealed no difference between adolescents who brushed twice daily or more and those who brushed less frequently. Oral health behaviors may be closely related to other dimensions of health and socio-economic development of an individual. A recent study from Saudi Arabia reported that ‘good’ lifestyle practices were significantly associated with dental behaviors [40]. A large nationally representative Finnish study [41] reported that adolescents with infrequent toothbrushing were more likely to be involved with problem behaviors, such as smoking and alcohol use. The same study also reported that adolescents with low toothbrushing frequency had the lowest educational attainment in their early middle age. In the current study, healthy adolescents had significantly higher odds of toothbrushing twice or more daily as compared with adolescents with components of MetS. It may be hypothesized that healthy adolescents may have higher levels of motivation to improve personal attractiveness and a stronger desire to achieve social acceptance than their counterparts. These findings clearly indicated that oral hygiene habits are not only associated with systemic health, but also may influence other dimensions of an individual’s life. In a conceptual model developed by Aarø et al [42], it was reported that addictive behaviors (e.g., smoking) and healthy behaviors (e.g., oral hygiene, physical activity and healthy nutrition), were closely associated. Young people who take care of their teeth behave in ways promoting other dimensions of health as well. Unfortunately, dental behaviors have seldom been included in studies examining interrelationships between systemic health and well-being. The findings of this study add valuable information to the existing literature on the relationship between oral hygiene practices and systemic health.

In this study, adolescents with MetS components had higher levels of salivary CRP, insulin IL-6 and adiponectin compared to healthy subjects. Festa et al. [43] also reported similar findings, where positive correlations were observed between CRP and BMI, BP, triglycerides, cholesterol, plasma glucose and fasting insulin. Several studies reported that higher levels of circulating CRP and insulin were associated with metabolic disorders [44]. Most studies on MetS have used blood or serum for the estimation of biomarkers. This is quiet challenging, especially among the younger population. In this study, salivary biomarkers were used, which provided a relatively easy, non-invasive means of identifying inflammatory markers, which will aid in early diagnosis of individuals who are at risk for developing MetS [45].

Most of the features of MetS are associated with insulin resistance. Several factors contribute to insulin resistance, which include genetic factors, sedentary lifestyle, increased consumption of high calorie food, lack of adequate physical activity and sleep apnea [46]. The present study also sought to assess the role of diet and physical activity on the change of status in MetS. This study found that the frequency of dining-out was associated with the presence of MetS components. Physical activity levels did not show a similar association with MetS. However, adolescents who brushed their teeth two or more times a day had significantly higher physical activity levels compared with adolescents who brushed less frequently. Therefore, toothbrushing might be an indicator for a healthy lifestyle. Well established evidence suggests that the worldwide increase in people being overweight, and obesity is largely due to modifiable lifestyle factors such as decreased physical activity, eating out and increased consumption of energy dense foods with high saturated fat and refined carbohydrates [47]. Meta-analyses of interventional studies among children have revealed that dietary modification and increased physical activity can lead to weight loss and also improved cardiometabolic risk factors, such as dyslipidemia and hypertension [48].

This study had a few limitations: firstly, the toothbrushing practices were self-reported, so reporting bias was possible; secondly, no information about the time spent on brushing or the brushing technique were assessed, which could introduce information bias; thirdly, this was an observational study and we could not conclude whether the more frequent toothbrushing decreased MetS; and finally, a few unmeasured potential confounders may have biased our estimates of the association between toothbrushing habits and metabolic abnormalities as they could not be included in the model, such as the level of gingival inflammation. Future studies addressing these limitations are needed to further explore the relationship between oral hygiene practices and MetS among children and adolescents.

Several cross-sectional studies have indicated the associations between the low frequency of toothbrushing and the high prevalence of various components of MetS [17,49]. The cross-sectional design of these studies prevents any causal inferences. We were able to address some of the limitations of the previous studies by conducting this 5-year longitudinal study and adjusting for potential confounding factors. 

## 5. Conclusions

To conclude, this study found that toothbrushing and frequency of dining-out were associated with the presence of MetS components among adolescents. Adolescents with MetS components had higher levels of salivary CRP, insulin IL-6 and adiponectin compared to healthy subjects. Maintaining good oral hygiene by frequent daily toothbrushing may not only improve dental health but also the general health of children and adolescents.

## Figures and Tables

**Table 1:** Baseline characteristics of the participants in relation to frequency of toothbrushing.

Baseline Characteristics		Frequency of Brushing		
	Total	≥2 Times/Day	≤1 Time/Day	*p*-Value *
	N (%)	N (%)	N (%)	
Total	348 (100.0)	186 (53.4)	162 (46.6)	
**Age in years (mean ± SD)**				
T1 (Year: 2014)	12.06 ± 0.59	12.03 ± 0.60	12.09 ± 0.58	0.35
T2 (Year: 2019)	17.04 ± 0.59	17.02 ± 0.60	17.06 ± 0.57	0.39
**Sex**				
Male	168 (48.3)	77 (45.8)	91 (54.2)	0.01
Female	180 (51.7)	109 (60.6)	71 (39.4)	
**Mother’s education**				
Not completed grade level	38 (10.9)	20 (52.6)	18 (47.4)	0.33
Less than high school	33 (9.5)	24 (72.7)	9 (27.3)	
Completed secondary education	46 (13.2)	24 (52.2)	22 (47.8)	
Certificate/diploma	164 (47.1)	82 (50.0)	82 (50.0)	
University	19 (5.5)	10 (52.6)	9 (47.4)	
Master/PhD	48 (13.8)	26 (54.2)	22 (45.8)	
**Father’s education**				
Not completed grade level	34 (9.8)	21 (61.8)	13 (38.2)	0.11
Less than high school	57 (16.4)	25 (43.9)	32 (56.1)	
Completed secondary education	52 (14.9)	30 (57.7)	22 (42.3)	
Certificate/diploma	123 (35.3)	69 (56.1)	54 (43.9)	
University	25 (7.2)	9 (36.0)	16 (64.0)	
Master/PhD	57 (16.4)	32 (56.1)	25 (43.9)	
**Medical condition**				
No known medical condition	267 (76.7)	148 (55.4)	119 (44.6)	0.20
Pre-existing medical condition	81 (23.3)	38 (46.9)	43 (53.1)	
**Currently on medication**				
No	297 (85.3)	159 (53.5)	138 (46.5)	0.53
Yes	51 (14.7)	27 (52.9)	24 (47.1)	
**Father is diabetic**				
No	243 (69.8)	131 (53.9)	112 (46.1)	0.52
Yes	88 (25.3)	44 (50.0)	44 (50.0)	
**Mother is diabetic**				
No	298 (85.6)	161 (54.0)	137 (46.0)	0.83
Yes	37 (10.6)	19 (51.4)	18 (48.6)	
**Physical exercise ^#^**				
Poor (<50th percentile)	203 (58.3)	98 (48.3)	105 (51.7)	0.02
Good (≥50th percentile)	145 (41.7)	88 (60.7)	57 (39.3)	
**Diet pattern analysis ^§^**				
Dining-out				
Rarely (≤25th percentile)	86 (24.7)	49 (57.0)	37 (43.0)	0.30
Sometimes (26th to 50th percentile)	89 (25.6)	41 (46.1)	48 (53.9)	
Often (51st to 75th percentile)	87 (25.0)	45 (51.7)	42 (48.3)	
Frequently (≥76th percentile)	86 (24.7)	51 (59.3)	35 (40.7)	
**T1—Number of metabolic abnormalities**				
0 (Not obese AND not hypertensive)	107 (30.7)	66 (61.7)	41 (38.3)	0.12
1 (Obese OR hypertensive)	130 (37.4)	66 (50.8)	64 (49.2)	
2 (Obese AND hypertensive)	111 (31.9)	54 (48.6)	57 (51.4)	
**T1—Metabolic abnormality**				
High BMI ^†^ (Kg/m^2^)	209 (60.1)	103 (49.3)	106 (50.7)	0.06
High BP ^‡^ (mm Hg)	143 (41.1)	71 (49.7)	72 (50.3)	0.27
**T2—Number of metabolic abnormalities**				
0 (Not obese AND not hypertensive)	145 (41.7)	90 (62.1)	55 (37.9)	0.02
1 (Obese OR hypertensive)	165 (47.4)	77 (46.7)	88 (53.3)	
2 (Obese AND hypertensive)	38 (10.9)	19 (50.0)	19 (50.0)	
**T2—Metabolic abnormality**				
High BMI ^†^ (Kg/m^2^)	191 (54.9)	89 (46.6)	102 (53.4)	0.003
High BP ^‡^ (mm Hg)	50 (14.4)	26 (52.0)	24 (48.0)	0.47
**Status of metabolic abnormality at T1 and T2**				
(1) Group 1 (T1= ‘+’ and T2= ‘+’)	83 (23.9)	52 (62.7)	31 (37.3)	0.03
(2) Group 2 (T1= ‘+’ and T2= ‘−’)	24 (6.9)	14 (58.3)	10 (41.7)	
(3) Group 3 (T1= ‘−’ and T2= ‘+’)	62 (17.8)	38 (61.3)	24 (38.7)	
(4) Group 4 (T1= ‘−’ and T2= ‘−’)	179 (51.4)	82 (45.8)	97 (54.2)	

‘+’ = healthy; ‘−’ = obese OR hypertensive. ^#^ ‘poor’ is defined as having values below the median composite score, and ‘good’ is defined as having values equal to or above the median composite score. ^§^ Based on a composite score from performing the principal component analysis (PCA) for the eating characteristics; ^†^ body mass index > 25 Kg/M^2^; ^‡^ systolic or diastolic blood pressure above the 95th percentile; * chi-square test or independent sample *t*-test.

**Table 2:** Dental characteristics of the participants in relation to frequency of toothbrushing.

Dental Characteristics	Frequency of Brushing			
	Total	≥2 Times/Day	≤1 Time/Day	*p*-Value *
	N (%)	N (%)	N (%)	
Mean number of teeth (mean ± SD)	25.71 ± 2.62	25.65 ± 2.31	25.77 ± 2.94	0.67
Dental caries experience (% with decay and fillings) (mean ± SD)	12.96 ± 0.66	13.63 ± 0.84	12.19 ± 1.02	0.28
Untreated decay (% with decay only) (mean ± SD)	8.90 ± 0.55	8.97 ± 0.69	8.81 ± 0.88	0.88
**Last dental visit**				
Less than 6 months ago	180 (51.7)	114 (63.3)	66 (36.7)	<0.01
One year ago	58 (16.7)	24 (41.4)	34 (58.6)	
Two or more years ago	66 (19.0)	22 (33.3)	44 (66.7)	
Do not know	44 (12.6)	26 (59.1)	18 (40.9)	
**Reason for the last dental visit**				
Examination	50 (18.6)	30 (60.0)	20 (40.0)	0.11
Cleaning	94 (34.9)	55 (58.5)	39 (41.5)	
Pain	68 (25.3)	29 (42.6)	39 (57.4)	
Others	17 (6.3)	6 (35.3)	11 (64.7)	
Do not know	40 (14.9)	19 (47.5)	21 (52.5)	
**Type of dental clinic**				
Government	181 (52.0)	90 (49.7)	91 (50.3)	0.30
Private	138 (39.7)	78 (56.5)	60 (43.5)	
Do not know	29 (8.3)	18 (62.1)	11 (37.9)	
**Dental pain in the last year**				
Yes	83 (23.9)	47 (56.6)	36 (43.4)	0.08
No	242 (69.5)	122 (50.4)	120 (49.6)	
Do not know	23 (6.6)	17 (73.9)	6 (26.1)	
**Embarrassment due to teeth problems**				
No	205 (58.9)	107 (52.2)	98 (47.8)	0.59
Yes	143 (41.1)	79 (55.2)	64 (44.8)	

* Chi-square test or Independent sample *t*-test.

**Table 3:** Summary statistics for salivary biomarkers by brushing frequency.

Biomarkers		Frequency of Brushing		
	Total	≥2 Times/Day	≤1 Time/Day	*p*-Value *
(pg/mL)	Median (IQR)	Median (IQR)	Median (IQR)	
T1—CRP	367.9 (773.3)	305.8 (1066.9)	483.3 (577.0)	0.75
T2—CRP	347.3 (754.8)	275.3 (738.9)	417.3 (769.2)	0.09
T1—Leptin	162.3 (75.0)	162.3 (58.0)	104.1 (122.0)	0.05
T2—Leptin	181.6 (111.0)	181.2 (95.0)	181.2 (244.0)	0.16
T1—Insulin	416.3 (516.9)	451.7 (599.1)	348.5 (427.7)	0.57
T2—Insulin	284.0 (294.4)	279.4 (275.6)	317.5 (329.1)	0.14
T1—IL-6	8.2 (18.2)	8.3 (18.8)	7.9 (19.1)	0.86
T2—IL-6	3.9 (5.4)	3.7 (5.0)	4.1 (6.8)	0.23
T1—IL-8	2059.9 (1527.8)	1717.0 (1081.8)	2124.8 (3114.5)	0.31
T2—IL-8	474.5 (369.0)	479.6 (366.0)	463.4 (379.0)	0.68
T1—IL-10	1.6 (1.0)	1.6 (2.0)	1.6 (2.0)	0.65
T2—IL-10	0.5 (0.4)	0.5 (0.4)	0.5 (0.4)	0.24
T1—MCP	359.3 (237.2)	315.9 (200.8)	389.1 (440.6)	0.25
T2—MCP	308.4 (293.2)	299.8 (331.9)	315.5 (280.3)	0.49
T1—Adiponectin	6823.0 (10,286.5)	6504.8 (11,924.9)	7104.2 (8721.9)	0.88
T2—Adiponectin	4936.9 (6754.2)	4870.5 (6247.3)	5313.7 (7728.1)	0.73
T1—VEGF	1049.9 (607.8)	1108.8 (775.4)	1049.9 (376.3)	0.73
T2—VEGF	667.9 (464.8)	613.4 (474.5)	718.8 (447.1)	0.08

* Mann–Whitney U test. CRP—C-reactive protein; IL—interleukin; MCP—monocyte chemotactic protein; VEGF—vascular endothelial growth factor.

**Table 4:** Median and inter-quartile range (IQR) for salivary biomarkers at T1 and T2 for the metabolic categories.

Biomarkers	T1 (2014)					T2 (2019)				
(pg/mL)	Total	Group 1 (T1= ‘+’ and T2= ‘+’)	Group 2 (T1= ‘+’ and T2= ‘−’)	Group 3 (T1= ‘−’ and T2= ‘+’)	Group 4 (T1= ‘−’ and T2= ‘−’)	Total	Group 1 (T1= ‘+’ and T2= ‘+’)	Group 2 (T1= ‘+’ and T2= ‘−’)	Group 3 (T1= ‘−’ and T2= ‘+’)	Group 4 (T1= ‘−’ and T2= ‘−’)
	**Median (IQR)**					**Median (IQR)**				
CRP	367.97 (773.3)	327.53 (454.98)	163.38 (84.3)	263.27 (1131.0)	597.59 (1038.0)	347.29 (754.8)	**223.69 (416.5) ^e^**	352.23 (633.8)	218.47 (360.4)	**520.17 (1137.3) ^e^**
Leptin	162.30 (85.0)	162.3 (0.0)	104.10 (0.0)	104.10 (0.0)	162.30 (66.0)	181.22 (111.0)	181.86 (107.0)	203.36 (118.0)	182.49 (87.0)	167.55 (111.0)
Insulin	416.64 (516.6)	**135.94 (171.2) ^f^**	310.09 (213.1)	**153.02 (498.0) ^a^**	**581.97 (396.8) ^f^**	284.00 (294.2)	**215.13 (218.9) ^g^**	309.82 (253.2)	**262.07 (208.1) ^a^**	**334.31 (393.9) ^g^**
IL-6	8.26 (18.21) *	**4.28 (8.2) ^h^**	7.39 (1.73)	**10.09 (48.1) ^b^**	**21.46 (84.9) ^h^**	3.98 (5.47) *	3.73 (6.7)	3.98 (6.5)	**4.59 (5.7) ^b^**	3.98 (3.6)
IL-8	2059.86 (1527.8) *	2059.86 (3359.5)	2209.01 (227.6)	988.47 (1872.2)	2050.89 (1517.1)	474.47 (369.0) *	463.59 (396.0)	436.59 (565.0)	388.21 (311.0)	486.09 (378.0)
IL-10	1.63 (1.0)	1.78 (2.0)	1.47 (0.0)	3.84 (7.0)	1.39 (1.0)	0.49 (0.37)	0.37 (0.39)	0.49 (0.57)	0.50 (0.55)	0.50 (0.44)
MCP	359.32 (237.2)	387.76 (204.1)	865.24 (239.3)	415.69 (554.0)	**332.40 (189.7) ^c^**	308.41 (293.2)	278.27 (277.2)	363.01 (334.9)	345.84 (391.4)	**306.10 (268.5) ^c^**
Adiponectin	6823.00 (10,286.5) *	**5162.43 (4884.8) ^i^**	6618.50 (4635.0)	13,513.48 (40,963.4)	**14,767.55 (14,438.5) ^i^**	4936.86 (6754.2) *	5570.02 (6758.8)	3984.53 (5866.8)	6134.31 (8810.2)	4552.64 (6537.7)
VEGF	1049.94 (607.8) *	1284.74 (680.9)	1281.24 (0.0)	1017.23 (732.4)	**971.25 (622.5) ^d^**	667.90 (464.8) *	706.90 (371.4)	617.80 (645.4)	586.18 (536.7)	**685.75 (472.1) ^d^**

‘+’ = healthy; ‘−’ = obese OR hypertensive. Group 1 (T1 = ‘+’ and T2 = ‘+’); Group 2 (T1 = ‘+’ and T2 = ‘−’); Group 3 (T1 = ‘−’ and T2 = ‘+’); Group 4 (T1 = ‘−’ and T2 = ‘−’). CRP—C-reactive protein; IL—interleukin; MCP—monocyte chemotactic protein; VEGF—vascular endothelial growth factor. * Significant difference in the overall median scores of each biomarker between T1 and T2 (*p* < 0.05; Wilcoxon signed ranks test). ^a,b,c,d^ Significant difference in the levels of biomarkers between the metabolic groups at T1 and T2 (*p* < 0.05; Wilcoxon signed ranks test). ^e,f,g,h,i^ Significant difference in the levels of biomarkers within the metabolic groups (*p* < 0.05; Kruskal–Wallis test). Significant associations are shown in bold fonts.

**Table 5:** Multinomial logistic regression models for the association between toothbrushing and change in metabolic syndrome after adjusting the covariates.

Variables		Model 1 ^a^			Model 2 ^b^			Model 3 ^c^		
	Group 4 (T1 = ‘−’ and T2 = ‘−’)	Group 1 (T1 = ‘+’ and T2 = ‘+’)	Group 2 (T1 = ‘+’ and T2 = ‘−’)	Group 3 (T1 = ‘−’ and T2 = ‘+’)	Group 1 (T1 = ‘+’ and T2 = ‘+’)	Group 2 (T1 = ‘+’ and T2 = ‘−’)	Group 3 (T1 = ‘−’ and T2 = ‘+’)	Group 1 (T1 = ‘+’ and T2 = ‘+’)	Group 2 (T1 = ‘+’ and T2 = ‘−’)	Group 3 (T1 = ‘−’ and T2 = ‘+’)
		**OR (95% CI)**			**OR (95% CI)**			**OR (95% CI)**		
Toothbrushing										
≥2 times/day	Reference	**1.990 (1.148–3.450)**	1.664 (0.670–4.133)	1.910 (1.040–3.505)	**2.048 (1.148–3.653**)	2.082 (0.806–5.374)	**2.159 (1.125–4.141)**	**1.987 (1.062–3.715)**	2.043 (0.740–5.641)	**2.024 (1.027–3.990)**
≤1 time/day	Reference	Reference	Reference	Reference	Reference	Reference	Reference	Reference	Reference	Reference
**Gender**										
Male	Reference	0.816 (0.467–1.425)	1.316 (0.521–3.322)	1.086 (0.588–2.009)	0.772 (0.428–1.390)	1.340 (0.497–3.612)	0.860 (0.444–1.668)	0.766 (0.407–1.443)	1.346 (0.466–3.888)	0.865 (0.435–1.728)
Female	Reference	Reference	Reference	Reference	Reference	Reference	Reference	Reference	Reference	Reference
**Physical activity**										
Poor (<50th percentile)	Reference	1.150 (0.651–2.030)	1.018 (0.403–2.574)	1.205 (0.643–2.257)	1.142 (0.631–2.065)	1.076 (0.402–2.881)	1.248 (0.643–2.421)	1.160 (0.621–2.166)	1.032 (0.365–2.919)	1.299 (0.656–2.571)
Good (≥50th percentile)	Reference	Reference	Reference	Reference	Reference	Reference	Reference	Reference	Reference	Reference
**Frequency of dining-out pattern analysis**										
Rarely (<25th percentile)	Reference	0.695 (0.318–1.521)	**0.142 (0.030–0.683)**	0.961 (0.423–2.187)	0.687 (0.301–1.568)	**0.111 (0.022–0.563)**	0.978 (0.406–2.352)	0.671 (0.281–1.600)	**0.087 (0.016–0.486)**	1.044 (0.429–2.576)
Sometimes (25th to 50th percentile)	Reference	0.998 (0.470–2.121)	0.376 (0.119–1.183)	0.814 (0.345–1.923)	0.846 (0.381–1.880)	0.309 (0.091–1.053)	0.918 (0.361–2.330)	0.888 (0.382–2.062)	0.279 (0.075–1.040)	0.942 (0.364–2.439)
Frequently (51st to 75th percentile)	Reference	0.956 (0.447–2.045)	0.380 (0.121–1.197)	0.877 (0.375–2.051)	0.922 (0.415–2.050)	0.327 (0.096–1.117)	0.906 (0.362–2.266)	0.941 (0.408–2.170)	0.412 (0.112–1.512)	0.929 (0.367–2.356)
Very frequently (>75th percentile)	Reference	Reference	Reference	Reference	Reference	Reference	Reference	Reference	Reference	Reference

‘+’ = healthy; ‘−’ = obese OR hypertensive; Group 1 (T1 = ‘+’ and T2 = ‘+’); Group 2 (T1 = ‘+’ and T2 = ‘−’); Group 3 (T1 = ‘−’ and T2 = ‘+’); Group 4 (T1 = ‘−’ and T2 = ‘−’); ^a^ Model 1—basic model adjusted for gender, physical activity and diet. ^b^ Model 2—additionally adjusted for maternal/paternal education, maternal/paternal diabetic status and medical history (currently on medication and any known medical condition). ^c^ Model 3—additionally adjusted for dental characteristics (mean number of teeth, last dental visit, dental pain in the last year and embarrassment due to teeth problems). Significant associations are shown in bold fonts.

## Data Availability

Data used in this study is available from the corresponding author upon reasonable request.
